# Deciphering Molecular Determinants Underlying *Penicillium digitatum’s* Response to Biological and Chemical Antifungal Agents by Tandem Mass Tag (TMT)-Based High-Resolution LC-MS/MS

**DOI:** 10.3390/ijms23020680

**Published:** 2022-01-08

**Authors:** Lucía Citores, Mariangela Valletta, Vikram Pratap Singh, Paolo Vincenzo Pedone, Rosario Iglesias, José Miguel Ferreras, Angela Chambery, Rosita Russo

**Affiliations:** 1 Department of Biochemistry and Molecular Biology and Physiology, Faculty of Sciences, University of Valladolid, E-47011 Valladolid, Spain; lucia.citores@uva.es (L.C.); riglesia@bio.uva.es (R.I.); josemiguel.ferreras@uva.es (J.M.F.); 2 Department of Environmental, Biological and Pharmaceutical Sciences and Technologies, University of Campania Luigi Vanvitelli, 81100 Caserta, Italy; mariangela.valletta@unicampania.it (M.V.); vikrampratap.singh@unicampania.it (V.P.S.); paolovincenzo.pedone@unicampania.it (P.V.P.)

**Keywords:** beetin 27 (BE27), *Penicillium digitatum*, proteomics, ribosome-inactivating proteins (RIPs), ribotoxins, citrus green mold

## Abstract

*Penicillium digitatum* is a widespread pathogen responsible for the postharvest decay of citrus, one of the most economically important crops worldwide. Currently, chemical fungicides are still the main strategy to control the green mould disease caused by the fungus. However, the increasing selection and proliferation of fungicide-resistant strains require more efforts to explore new alternatives acting via new or unexplored mechanisms for postharvest disease management. To date, several non-chemical compounds have been investigated for the control of fungal pathogens. In this scenario, understanding the molecular determinants underlying *P. digitatum’s* response to biological and chemical antifungals may help in the development of safer and more effective non-chemical control methods. In this work, a proteomic approach based on isobaric labelling and a nanoLC tandem mass spectrometry approach was used to investigate molecular changes associated with *P. digitatum’s* response to treatments with α-sarcin and beetin 27 (BE27), two proteins endowed with antifungal activity. The outcomes of treatments with these biological agents were then compared with those triggered by the commonly used chemical fungicide thiabendazole (TBZ). Our results showed that differentially expressed proteins mainly include cell wall-degrading enzymes, proteins involved in stress response, antioxidant and detoxification mechanisms and metabolic processes such as thiamine biosynthesis. Interestingly, specific modulations in response to protein toxins treatments were observed for a subset of proteins. Deciphering the inhibitory mechanisms of biofungicides and chemical compounds, together with understanding their effects on the fungal physiology, will provide a new direction for improving the efficacy of novel antifungal formulations and developing new control strategies.

## 1. Introduction

Citrus fruits (genus *Citrus* in family Rutaceae), comprising oranges, grapefruit, mandarins, limes and lemons, are among the most widespread fruit crop worldwide, highly enriched with components beneficial to human health. The great economic value of the citrus fruits market is also based on properties that largely rely on factors affecting both external and internal quality. In the fresh market, these aspects are both important since external quality influences the initial purchasing decision, while internal quality determines consumption on repeat sale. Attractive external quality characteristics for consumers include size, shape, deep peel colour and smooth and shiny appearance [1]. At the same time, the main causes of rejects include weight loss, presence of bruises and cuts, symptoms of mould and decay and colour changes. In addition, while no bacterial postharvest diseases of commercial importance have been reported, citrus fruits are highly susceptible to fungi infection [2]. Among them, the green mould and blue mould, caused by *Penicillium digitatum* and *Penicillium italicum*, respectively, represent the two most important diseases in all citrus production during fruits postharvest handling procedures [3,4]. In particular, the postharvest green mould is the main factor affecting citrus fruit decay, leading to huge economic losses worldwide every year and accounting for up to 90% of the total citrus postharvest losses, especially in subtropical climates [4].

Synthetic fungicides played a key role in crop protection for the control of the citrus postharvest green mould. However, fungicide resistance represents a critical issue worldwide, with still limited approaches for effective disease management [5]. Thus, developing efficient alternative approaches for counteracting postharvest diseases of citrus fruits caused by Penicillium species has become an active field of research. Several promising biological control strategies, including the use of antagonistic microorganisms, the application of naturally-derived bioactive compounds and the induction of natural resistance, have been proposed as potential alternatives to synthetic fungicides for the control of citrus diseases [2].

These approaches rely on naturally occurring agents such as several secreted proteins from fungi endowed with antiviral, antibacterial, antifungal, and insecticidal activities such as the antifungal protein (AFP) and the ribotoxin α-sarcin from *Aspergillus giganteus* [6,7,8,9]. Recently, it was reported that α-sarcin, traditionally considered toxic only to animal cells, displays a strong antifungal activity against *Penicillium digitatum* for its ability to enter into the cytosol and inactivate the ribosomes, thus killing cells and arresting fungus growth [10]. Ribotoxins such as α-sarcin are rRNA endonucleases (EC 4.6.1.23) that catalyse the cleavage of the phosphodiester bond on the 3′ side of the G4325 residue from the rat large rRNA. This nucleotide is located in the Sarcin Ricin Loop (SRL) that is involved in the binding of the elongation factor to the ribosome [10]. In addition, several plant-derived proteins such as the ribosome-inactivating proteins (RIPs) have been extensively studied for their antiviral, antifungal and insecticidal activity mediated by the inhibition of protein synthesis [11,12]. RIPs belong to a class of enzymes (EC 3.2.2.22) that exhibits rRNA N-glycosylase activity. This activity prevents protein synthesis by causing the release of a specific adenine residue in the SRL of the large rRNA. A marked antifungal activity against the green mould *Penicillium digitatum* has been described for the apoplastic protein beetin 27 (BE27), a RIP isolated from sugar beet (*Beta vulgaris* L.) leaves. BE27 is able to enter into the cytosol and kill cells, thus arresting the growth of the fungus at a concentration much lower than that present in the apoplast [13].

In this work, the molecular determinants of *Penicillium digitatum* response to the ribotoxin α-sarcin and the RIP BE27 were investigated. We further evaluated protein changes following treatment with the commonly used fungicide thiabendazole (TBZ), a microtubule-destabilizing drug that inhibits mitosis [14], with the aim to compare *P. digitatum* response to biological and chemical fungicides. An advanced quantitative proteomic approach based on Tandem Mass Tag (TMT) isobaric labelling and nano-liquid chromatography coupled with high resolution tandem mass spectrometry (nanoLC MS/MS) was used to identify and quantify the expression levels of proteins from treated and untreated *P. digitatum* mycelia. 

Collectively, we identified candidate proteins potentially associated with α-sarcin, BE27 and TBZ treatment outcomes in *P. digitatum*. These were mainly involved in cell wall degradation and fungal morphogenesis, stress response, antioxidant and detoxification mechanisms and metabolic pathways. Although a similar trend in protein changes was driven by the three antifungal treatments, a distinct regulation in response to α-sarcin and BE27 treatments was also observed for a subset of proteins, paving the way to understanding the molecular determinants underlying *P. digitatum* response to biological and synthetic antifungal agents.

## 2. Results

### 2.1. Effects of BE27, α-Sarcin and TBZ Treatments on the Growth and Morphogenesis of P. digitatum

**Figure 1:**
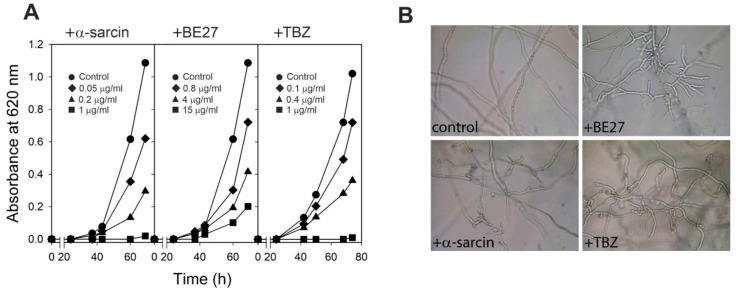
Antifungal activity of α-sarcin, BE27 and TBZ against *Penicillium digitatum*. (**A**) Conidia of *P. digitatum* were grown at 26 °C in PDB medium in the presence of different concentrations of α-sarcin (**left**), BE27 (**centre**) and TBZ (**right**). Fungal growth was measured as an increase in absorbance at 620 nm. The curves represent for α-sarcin treatment, (●) buffer control, (◆) 0.05 μg/mL (▲) 0.2 μg/mL and (■) 1 μg/mL; for BE27 treatment (●) buffer control, (◆) 0.8 μg/mL (▲) 4 μg/mL and (■) 15 μg/mL; and for TBZ treatment (●) buffer control, (◆) 0.1 μg/mL (▲) 0.4 μg/mL and (■) 1 μg/mL. (**B**) Morphological changes of *P. digitatum* mycelium exposed to α-sarcin, BE27 and TBZ. *P. digitatum* mycelium was grown in the absence (control) or in the presence of 0.2 μg/mL α-sarcin, 4 μg/mL BE27 and 0.4 μg/mL TBZ. After 60 h incubation, samples were visualized using light microscopy at 200× magnification.

A strong activity against *P. digitatum* has been recently reported following treatments with two inhibitors of protein synthesis, BE27 and α-sarcin [10,13], suggesting a potential application of these proteins as antifungal agents in nature. We therefore performed a preliminary investigation to evaluate the effects of different treatments with α-sarcin, BE27 and TBZ on the growth and morphogenesis of *P. digitatum*. As shown in Figure 1A, α-sarcin, BE27 and TBZ reduced the fungal growth in a concentration-dependent manner. Thus, 0.2 μg/mL α-sarcin, 4 μg/mL BE27 and 0.4 μg/mL TBZ resulted in approximately 60% growth inhibition when the fungal conidia were treated for about 70 h. By using light microscopy, morphological changes were visualized within treated cultures with respect to the untreated control (Figure 1B). As expected, treated cultures revealed aborted hyphal branches with respect to the regular and homogeneous hyphae observed within the control, thus confirming that both proteins and TBZ exerted a strong effect on the growth of *P. digitatum* at concentrations resulting in 60% growth inhibition.

### 2.2. Quantitative Proteomic Analysis of P. digitatum in Response to α-Sarcin, BE27 and TBZ Treatments

**Figure 2:**
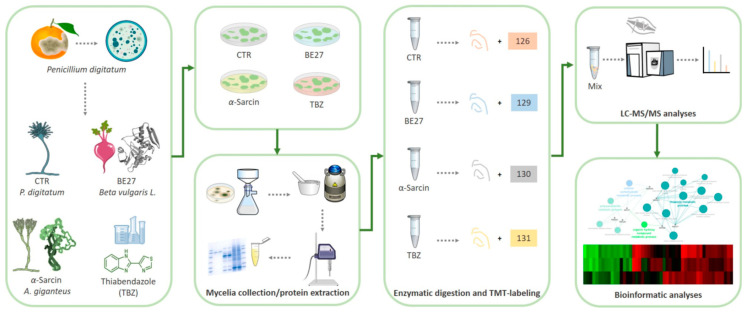
Schematic workflow applied for proteomic analysis of *P. digitatum* treated with fungicide compounds (i.e., α-sarcin, BE27 and TBZ). Following treatments, mycelia were collected and grounded in a ceramic mortar with liquid nitrogen. For Tandem Mass Tag (TMT) isobaric labelling, proteins extracted by sonication were digested into peptides and labelled with TMT isobaric stable isotope tags. After sample mixing, peptides were analysed by LC-ESI-MS/MS. In MS1, the peptides appear as a single precursor. When fragmented during MS2, in addition to the normal fragment ions, the reporter regions dissociated to produce ion signals, which provided accurate quantitative information regarding the relative amount of the peptide in the samples.

**Figure 3:**
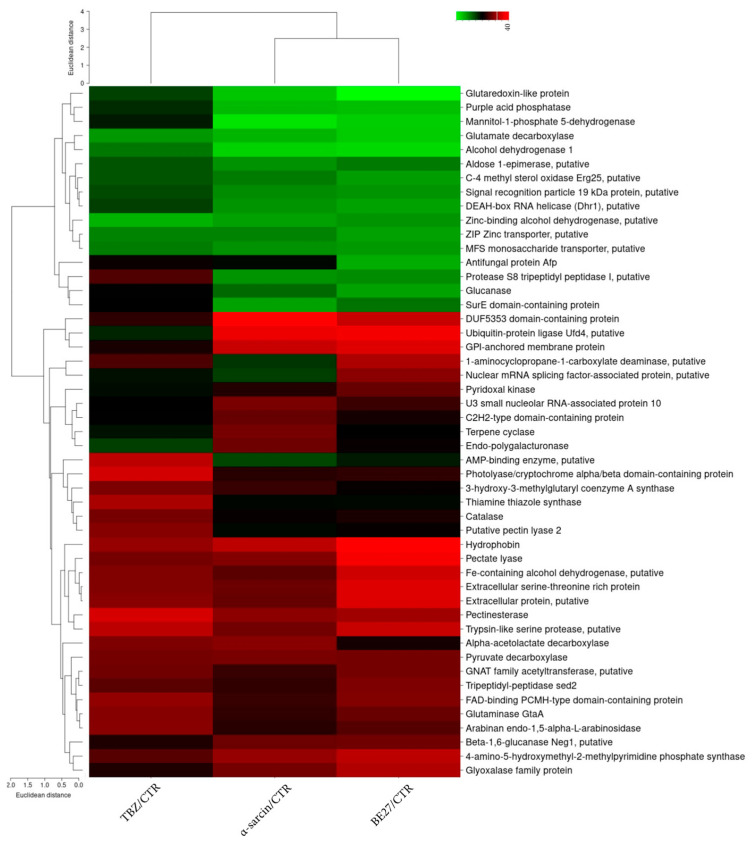
Heatmap representing the log2 fold-change values of differentially expressed proteins in α-sarcin, BE27- and TBZ-treated vs. untreated (CTR) *P. digitatum* samples. Down-regulated and up-regulated proteins are coloured in green and red, respectively.

To identify differentially expressed proteins upon α-sarcin, BE27, and TBZ treatments, a comparative proteomic analysis was performed on treated and untreated *P. digitatum* mycelia. A quantitative proteomic approach based on TMT isobaric labelling and nano-liquid chromatography coupled with high-resolution MS/MS analysis was adopted. A schematic workflow of the sample preparation and labelling procedure is shown in Figure 2. A concentration of about 90% unique peptides was used for protein quantification, assuring the high efficiency of peptide labelling. By MS/MS and database search, we identified and quantified 1709 non-redundant proteins with more than one peptide in at least two out of three injections with very low CV% values, attesting to the high reproducibility of the analysis (Supplementary Table S1 and Supplementary Figure S1). An overview of the quantitative analysis on unfiltered dataset is shown in the volcano plots reporting −Log10 *p*-values versus Log2 fold-change values of TBZ, BE27 and α-sarcin treatments in *P. digitatum* with respect to control (Supplementary Figure S2). Following data filtering (see paragraph 4.6), out of the identified proteins, we then extracted a subset of 49 proteins whose relative expression levels changed 1.5-fold or more (in any direction) in at least one treatment condition with respect to untreated *P. digitatum* (Table 1). These proteins were clustered in the heatmap shown in Figure 3 and included a subset of proteins whose levels changed in the same directions under different treatments (i.e., up- or down-regulated). Despite a similar response to different treatments, the comparative analysis of protein fold changes also revealed a distinctive response of *P. digitatum* to the protein toxins with respect to TBZ, suggesting the occurrence of both common and specific regulation responses depending on the antifungal treatment.

### 2.3. Categorization of Differentially Expressed Proteins 

**Figure 4:**
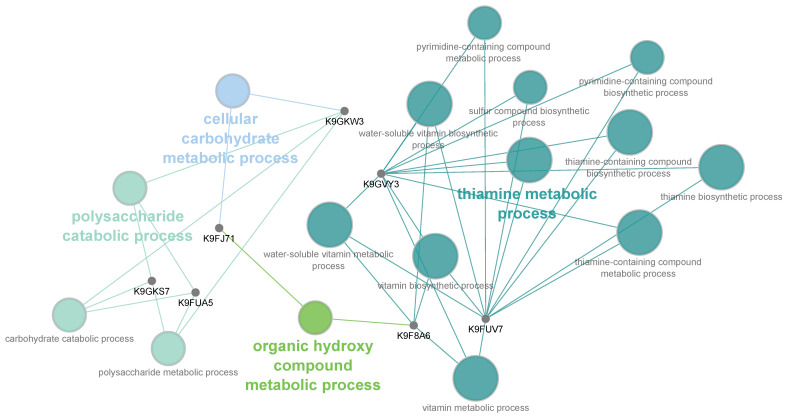
Functionally grouped network of enriched molecular function categories for a subset of differentially expressed proteins generated using the ClueGO Cytoscape plug-in. The proportion of shared proteins between terms was evaluated using kappa statistics. GO terms are depicted as nodes whose size represents the term enrichment significance.

**Figure 5:**
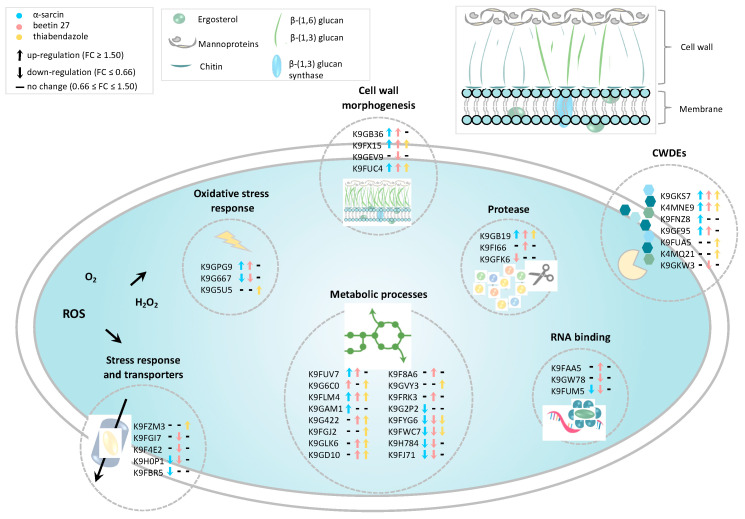
Overview of proteins modulated upon antifungal treatments. UniProt accession numbers and protein regulation levels are reported for each treatment (see arrow colours: light blue, α-sarcin; pink, beetin 27; yellow, thiabendazole).

A major challenge in the high-throughput identification (conducted by means of MS) and subsequent data analysis of proteins for non-model species is represented by the lack of annotation of protein sequences. Indeed, besides the availability of biological curated protein databases, bioinformatic tools for data processing and interpretation have become essential in MS-based proteomic analyses. By using the ClueGO Cytoscape plug-in implemented with *P. digitatum* taxa from UniProtKB, differentially expressed proteins were mapped on a clustered network showing that several nodes are related to metabolic processes including thiamine biosynthesis and polysaccharide metabolism (Figure 4). Overall, both bioinformatic and manual categorization approaches converge on the involvement of many differentially expressed proteins in biological processes related to cell wall-degrading enzymes (CWDEs), fungal morphogenesis, metabolic processes, the activation of antioxidant and detoxification mechanisms and the stress response to treatments (Figure 5). 

### 2.4. Cell Wall-Degrading Enzymes (CWDEs) and Fungal Morphogenesis

Several differentially expressed proteins were involved in fungal morphogenesis and cell wall degradation. Hydrophobin (AC: K9FX15), known to be involved in many fungal morphogenetic processes, including sporulation, fruit body development and infection structure formation, was found to be significantly upregulated in all three treatments with the higher regulation fold changes detected upon treatments with protein toxins (2.0 and 2.7 for α-sarcin and BE27, respectively). An additional protein belonging to the family of GPI-anchored proteins, required for cell wall biogenesis and morphogenesis in fungi, was also differentially modulated following treatments. Indeed, the GPI-anchored membrane protein (AC: K9GB36) was upregulated in α-sarcin and BE27 treatment, whereas, in TBZ treatment, no significant changes were revealed for the same protein. In the BE27 sample alone, a downregulation of the C-4 methyl sterol oxidase Erg25 (AC: K9GEV9), an essential enzyme involved in ergosterol biosynthesis, was also observed. Many differentially expressed proteins were classified as cell wall-degrading enzymes (CWDEs) traditionally associated with the fungal virulence and plant infection severity. Among these, Pectinesterase (AC: K9GKS7) and Pectate lyase (AC: K9GKS7) were commonly upregulated following all the three treatments. By contrast, a different response was found for the Endo-polygalacturonase (AC: K9FNZ8), which was only upregulated in the α-sarcin-treated sample. Similarly, only the Beta-1,6-glucanase Neg1 (AC: K9GF95) was upregulated following both protein treatments. Interestingly, another Glucanase (AC: K9GKW3), catalysing the hydrolysis of (1,4-)-beta-d-glucosidic linkages in cellulose, was only downregulated in a *P. digitatum* sample treated with BE27. A similar response was observed for the Tripeptidyl-peptidase sed2 (AC: K9FI66), upregulated only in the BE27-treated sample, while the Protease S8 tripeptidyl peptidase I (AC: K9FI66) was commonly downregulated by both treatments with protein toxins. Noteworthy, proteases act as pivotal hydrolytic enzymes engaged by fungal pathogens for infection of host plants. Of potential relevance for plant infection, these findings suggest that α-sarcin and BE27 may elicit a similar and a peculiar response towards the maintenance of *P. digitatum* mycelium morphogenesis and cell wall integrity with respect to the synthetic TBZ antifungal agent.

### 2.5. Antifungal Agents Affect Penicillium digitatum Metabolic Processes 

A number of proteins related to carbohydrate, lipid and amino acid metabolism were found to be differentially regulated by antifungal treatments. A subset of these enzymes, belonging to the class of oxidoreductases, were downregulated following α-sarcin and BE27 treatments such as Alcohol dehydrogenase-1 (AC: K9H784), Zinc-binding alcohol dehydrogenase (AC: K9FYG6) and Mannitol-1-phosphate dehydrogenase (AC: K9FJ71). By contrast, the enzyme Pyruvate decarboxylase that catalyses the formation of acetaldehyde on the pathway of ethanol synthesis was upregulated following both protein toxins and TBZ treatments (AC: K9FLM4). This enzyme uses thiamine diphosphate (TPP) as an essential cofactor. Accordingly, our data revealed, for BE27 and TBZ, respectively, an up-regulation of enzymes involved in Thiamine biosynthesis such as Pyridoxal kinase (AC: K9F8A6) and Thiamine thiazole synthase (AC: K9GVY3). Supporting these data, also the enzyme 4-amino-5-hydroxymethyl-2-methylpyrimidine phosphate synthase (HMP-P Synthase; AC: K9FUV7), responsible for thiamine biosynthesis, was found to be up-regulated in all three anti-fungal treatments. 

### 2.6. Antioxidant and Detoxification Mechanisms and Stress Response

Our proteomic analysis further revealed a differential modulation of proteins related to reactive oxygen species (ROS) homeostasis, such as Catalase (AC: K9G5U5), upregulated in TBZ treatment and Glyoxalase family protein (AC: K9GPG9), upregulated upon α-sarcin and BE27 treatments. Moreover, for the Glutaredoxin-like protein (AC: K9G667), a glutathione-dependent oxidoreductase playing a role in redox homeostasis, a significant downregulation was observed following treatments with protein toxins. Collectively, these results point to a differential modulation of redox system players by anti-fungal treatments. Another important class of differentially regulated proteins was related to stress response and detoxification. Among these, key mediators of fungicide resistance were downregulated following treatments with α-sarcin and BE27 such as the MFS transporter (AC: K9H0P1). Another transporter involved in zinc homeostasis (i.e., the ZIP Zinc transporter, AC: K9FYG6) was only downregulated upon BE27 treatment as well as the Antifungal protein AFP (AC: K9FGI7), a small cysteine-rich protein endowed with a well-known specific antifungal activity.

## 3. Discussion

Over the past decade, the technological advances in mass spectrometry (MS)-based proteomics, together with the availability of *P. digitatum* and other fungi genome sequence information, enormously increased the potential to characterize the molecular changes occurring within pathogenic fungi in response to treatments with novel biofungicides. Recently, a label-free quantitative proteomic analysis was performed on *Fusarium oxysporum* f. sp. *cucumerinum* mycelia following treatment with canthin-6-one, an alkaloid compound extracted from *Ailanthus altissima*, with the aim of investigating the molecular mechanisms underlying the antifungal properties of this molecule [15]. A similar approach was applied to decipher the effects of the antifungal peptide ETD151, an analogue of the antifungal insect defensin heliomicin, on the phytopathogenic fungus *Botrytis cinerea* [16]. In addition, an iTRAQ-based LC-MS/MS analysis was used to evaluate the proteomic profiling of *B. cinerea* in response to wuyiencin, produced by *Streptomyces albulus* subsp. *wuyiensis*, widely used as an antifungal agent in agriculture [17]. The high-throughput capability of mass spectrometry analyses was exploited to evaluate proteomic changes occurring within *Penicillium* species following treatment with plant-derived pesticides. Recently, proteomic changes occurring following treatment of *P. expansum* with chitosan, used as a promising alternative for postharvest diseases management, were investigated by two-dimensional electrophoresis (2-DE) coupled to MALDI-TOF MS analysis [18]. A comparative analysis of both mycelial and extracellular proteomes of *Penicillium janczewskii* was performed to evaluate the impact on the fungus metabolism of the labdanolic acid, a terpenoid from *Cistus ladanifer* [19]. Moreover, an iTRAQ-based high resolution MS approach was applied for the determination of proteomic alterations in *P. expansum* spores under decanal stress [20]. Similarly, *Penicillium digitatum* proteome changes were evaluated in response to the antifungal extract produced by *Streptomyces lavendulae* strain X33 with the aim of exploring the intrinsic molecular mechanism of the bacterial extract on the fungus [21].

The present study features the proteomic analysis of *P. digitatum* to probe the molecular mechanism underling the effects of α-sarcin and BE27, two inhibitors of protein synthesis from *A. giganteus* and *B*. *vulgaris*, respectively, as biofungicides. We also evaluated differences in responses to protein toxin treatments with respect to thiabendazole (TBZ), a commonly used synthetic antifungal agent for *P. digitatum*. Our accurate quantitative analysis relied on an isobaric labelling strategy and high-resolution mass spectrometry analysis, for the first time used to investigate the molecular determinants involved in responses to natural and synthetic fungicides. 

We focused primarily on differentially expressed proteins potentially impairing *P. digitatum* growth and virulence such as cell wall-degrading enzymes (CWDEs) and proteases. In phytopathogenic fungi, an arsenal of catalytic proteins supports nutrient acquisition, conidial formation, substrate colonization and host invasion [22]. We found several CWDEs and proteases that were differentially regulated upon protein toxins and TBZ antifungal treatments such as pectinesterases, pectate lyases, polygalacturonases, glucanases and peptidases. The importance of CWDEs in the virulence and pathogenesis of *P. digitatum* has been reported during the infection of oranges [23] and on postharvest citrus [24].

On the pathogen side, we observed an upregulation of CWDEs belonging to the classes of pectinesterases and pectate lyases when treated with both biofungicides and TBZ, while some differences among treatments were related to the expression levels of polygalacturonases and glucanases as well as some peptidases. These results suggest that α-sarcin and BE27 toxins may trigger a specific response in modulating cell wall integrity. In this framework, the proteins involved in cell wall morphogenesis and remodelling were also differentially regulated, such as Hydrophobin and GPI-anchored membrane protein. Hydrophobins are surface-active proteins produced by filamentous fungi. They have a role in fungal growth as structural components and fungi’s interaction with their environment. They are essential for aerial growth and the attachment of fungi to solid supports. Spores of filamentous fungi are also covered by Hydrophobins that renders the conidial surface hydrophobic and wet-resistant [25]. In our experimental model, the upregulation of Hydrophobin may, thus, potentially also affect the observed effects on mycelium growth and morphogenesis. Other proteins that are functionally involved in maintaining cell wall integrity, such as GPI-anchored membrane proteins [26] and C-4 methyl sterol oxidase Erg25 [27,28], were differentially regulated by both protein toxins or by BE27 alone, respectively. These sources of evidence support the idea that toxins could affect the maintenance of the *P. digitatum* plasma membrane’s stability and fluidity.

Some proteins that are differentially regulated by antifungal treatments were related to energetic metabolism and amino acid metabolism, pointing to a global metabolic reprogramming during *P. digitatum’s* response to antifungal treatments. A subset of these proteins is involved in the biosynthesis of vitamins and cofactors such as thiamine (Vitamin B1) and its precursor thiazole. These molecules are precursors of the active thiamine diphosphate (TPP), an essential coenzyme of several metabolic enzymes including Pyruvate decarboxylase, which was also upregulated following both protein toxins and TBZ treatments. These results implied that a response in the expression of key enzymes involved in thiamine biosynthesis is elicited, which is likely to counteract the metabolic stress triggered by the three anti-fungal agents. Interestingly, consistent with our results, thiamine metabolism was also reported to be affected by the antifungal extract of the *S. lavendulae* strain X33 on the mycelial growth of *P. digitatum* [21]. 

Our results also revealed that treatments with fungicides trigger a response in some *P. digitatum* mediators of the redox system and stress-response homeostasis, such as the upregulation of Catalase in response to TBZ. Catalase is a well-known antioxidant enzyme produced by fungi as an infection strategy [29]. In *P. digitatum*, it was suggested that Catalase eliminates hydrogen peroxide produced by the plant as a defence mechanism [30]. Besides the role in plant infection, in agreement with our findings, catalase was also upregulated in *P. digitatum* treated with the X33 antifungal extract [21]. Proteins related to antioxidant defence response were also differentially regulated in responses to chitosan in *P. expansum* [31]. In our experimental system, further changes in the expression levels of Glyoxalase and Glutaredoxin-like proteins were elicited by α-sarcin and/or BE27, indicating that toxins incubation promoted the expression of defence-related enzymes to modulate redox homeostasis. 

Another stress-response protein, downregulated following treatments with α-sarcin and BE27, was the MFS monosaccharide transporter. MFS transporters are involved in virulence by regulating the secretion of host-specific toxins or providing protection against plant defence components [32]. Together with ABC transporters, these carriers are the most important efflux pumps involved in fungal protection against fungicides [33,34]. More importantly, they were also reported to increase resistance to fungicides through their ability to transport a wide variety of compounds, such as toxic products [23]. 

BE27 treatment alone also downregulate the expression level of the small cysteine-rich protein Antifungal protein AFP, which is endowed with a specific antifungal activity [35,36]. *P. digitatum* genome sequencing allowed the identification of several potential AFP-like proteins [37]. Although AFP constitutive expression in filamentous fungi has a negative impact on their growth and virulence, the biological role of endogenous proteins remains unclear. In other filamentous fungi such as *A. niger* and *A. giganteus*, it was suggested that AFPs are molecules that are important for survival under nutrient limitation [38]. In order to trigger a strong defence response, they were also hypothesized to act as sensors, signalling or effector molecules for plasma membrane destabilization and subsequent cell lysis [38].

## 4. Materials and Methods 

### 4.1. Materials

All chemicals, including tosyl phenylalanyl chloromethyl ketone (TPCK)-treated trypsin, were obtained from Thermo Scientific, unless otherwise stated. Acetonitrile (CH_3_CN, Honeywell Riedel-de Haen), formic acid (FA) and LC-MS grade water (Honeywell Riedel-de Haen) were obtained from Fisher Scientific Italia (Rodano, Milano). The tandem mass tag (TMT) TMT-sixplex isobaric mass tagging kit was purchased from Thermo Fisher Scientific (Rockford, IL, USA). The strain of *P. digitatum* was isolated in our laboratory and typified by the Spanish Type Culture Collection (CECT), Valencia, Spain. TBZ was obtained from Sigma Aldrich (St Louis, MO, USA). α-sarcin was purchased from Santa Cruz Biotechnology (Santa Cruz, CA, USA). BE27 was purified as described [13,39,40].

### 4.2. Antifungal Treatments of P. digitatum

*P. digitatum* mycelia for mass spectrometry analyses were prepared from cultures grown in 25-mm six-well plates containing 1.7 mL of PDB medium inoculated with 3000 spores, obtained as described in studies carried out by Citores et al. 2016 [13], in the absence or presence of BE27, α-sarcin and TBZ at the concentrations of 4, 0.2 and 0.4 µg/mL, respectively. The plates were incubated at 26 °C on a horizontal shaker (40 rpm). Following *P. digitatum* growth for 4 days, the mycelia were harvested by filtration through filter paper under vacuum, extensively washed with sterile water, weighed, and stored at −80 °C. The experiments were performed in duplicate with six wells.

### 4.3. Antifungal Activity Measurements

Growth inhibitory assays of BE27, α-sarcin and TBZ against *P. digitatum* were performed in 96-well microtiter plates. Conidia of *P. digitatum* (100 spores/well), obtained as indicated by Citores et al. 2016 [13], were incubated at 26 °C in 150 μL of potato dextrose broth (PDB) medium in the presence of different concentrations of BE27, α-sarcin and TBZ. Fungal growth was monitored spectrophotometrically using a microtiter plate Multiskan EX reader (Thermo Scientific, Waltham, MA, USA) and microscopically (Motic AE31 inverted Microscope) after the incubation times described in Figure 1. One representative experiment of three experiments performed in triplicate is shown. 

### 4.4. Sample Preparation for Mass Spectrometry Analysis

Protein extraction from *P. digitatum* samples grown in the presence and in the absence of BE27, α-sarcin and TBZ was performed by grounding 1.5 g of pooled mycelia in a ceramic mortar with liquid nitrogen. Then, 1 g of the sample was transferred to an Eppendorf tube, suspended in 1 mL of ice-cold lysis buffer (100 mM Triethylammonium bicarbonate, TEAB, 1% SDS) and disrupted by two cycles of sonication at a 40% amplitude for 20 s on ice. Lysates were cleared by centrifugation at 16,000× *g* for 15 min at 4 °C (Allegra 64R, Beckman Coulter, Milan, Italy). Supernatants were transferred to new tubes, treated with 1 Unit of RQ1 DNase (Promega) for 1 h at room temperature, and the protein concentration was determined by using the Pierce BCA Protein assay kit (Thermo Scientific, Rockford, IL, USA). For each condition, equal amounts of proteins (100 µg in 100 µL of 100 mM TEAB) were reduced with 10 mM Tris-(2-carboxyethyl)-phosphine (TCEP) for 1 h at 55 °C and alkylated with 18 mM iodoacetamide by incubating samples for 30 min at room temperature in the dark. Proteins were then precipitated overnight by adding six volumes of prechilled acetone. Following centrifugation at 8000× *g* for 10 min at 4 °C, protein pellets were resuspended in 100 µL of 100 mM TEAB and digested overnight with MS grade trypsin (Pierce, Thermo Scientific, Rockford, IL, USA) at an enzyme/substrate ratio of 1:40 at 37 °C. The resulting peptide mixtures were chemically labelled with the TMT isobaric tags as previously reported [41,42] by using the following tags: 126, 129, 130 and 131 for control (CTR), BE27, α-sarcin and TBZ treatments, respectively. Briefly, 0.8 mg of TMT reagents in 41 µL of anhydrous acetonitrile were added to each sample. The reaction proceeded for 1 h and was then quenched for 15 min with hydroxylamine to a final concentration of 0.3%. The mixture of samples was then prepared by combining the four conditions at equal amounts. Mixed samples were dried under vacuum in a SpeedVac Vacuum (Savant Instruments, Holbrook, NY, USA). Then, samples were resuspended in 2% trifluoroacetic acid (TFA) at the final concentration of 1 µg/µL and centrifuged at 10,000× *g* for 15 min. Aliquots of the supernatant (20 µL) were diluted in 2% TFA at the final concentration of 0.4 µg/µL and analysed in triplicate (5 µL/injection) by high resolution nano-LC-tandem mass spectrometry for LC-MS analyses.

### 4.5. High-Resolution NanoLC−Tandem Mass Spectrometry

Aliquots of TMT-labelled samples (2 µg) were analysed by high-resolution nanoLC−tandem mass spectrometry using a Q-Exactive Orbitrap mass spectrometer equipped with an EASY-Spray nanoelectrospray ion source (Thermo Scientific, Rockford, IL, USA) and coupled to a Thermo Scientific Dionex UltiMate 3000RSLC nano system (Thermo Scientific, Rockford, IL, USA). The solvent composition was 0.1% formic acid in water (solvent A) and 0.1% formic acid in acetonitrile (solvent B). Peptides were loaded on a trapping PepMap™100 μCartridge Column C18 (300 μm × 0.5 cm, 5 μm, 100 Å) and desalted with solvent A for 3 min at a flow rate of 10 μL/min. After trapping, eluted peptides were separated on an EASY-Spray analytical column (50 cm × 75 μm ID PepMap RSLC C18, 2 μm, 100 Å) heated to 35 °C at a flow rate of 300 nL/min, applying the following gradient: 5% B for 3 min, from 5% to 27.5% B in 222 min, from 27.5% to 40% B in 10 min, and from 40% to 95% B in 1 min. Washing (95% B for 4 min) and re-equilibration (5% B for 24 min) steps were always included at the end of the gradient. Eluting peptides were analysed on the Q-Exactive mass spectrometer operating in positive polarity mode with a capillary temperature of 280 °C and a potential of 1.9 kV applied to the capillary probe [43]. The full MS survey scan resolution was set to 70,000 with an automatic gain control (AGC) target value of 3 × 10^6^ for a scan range of 375–1500 m/z and a maximum ion injection time (IT) of 60 ms. A mass (m/z) of 445.12003 was used as the lock mass. A data-dependent top-12 method was operated, during which high-energy collisional dissociation (HCD) spectra were obtained at 35,000 MS2 resolution with an AGC target of 1 × 10^5^ for a scan range of 200–2000 m/z, maximum IT of 120 ms, 1.6 m/z isolation width and normalized collisional energy (NCE) of 32. Precursor ions targeted for HCD were dynamically excluded for 30 s. Full scans and Orbitrap MS/MS scans were acquired in profile mode, whereas ion trap mass spectra were acquired in centroid mode. Charge state recognition was enabled by excluding unassigned and 1, 7, 8, >8 charged states. All data were acquired with Xcalibur 3.1 software (Thermo Scientific, Rockford, IL, USA). 

### 4.6. Protein Identification and Quantitation

For data processing, the acquired raw files were analysed with the Thermo Scientific Proteome Discoverer 2.4 software (Thermo Scientific, Rockford, IL, USA) using the SEQUEST HT search engine. The HCD MS/MS spectra were searched against the *Penicillium digitatum* UniProtKB_TrEMBL database (version: 2020-10-7; number of entries: 10,053 sequences) assuming trypsin (Full) to be the digestion enzyme and with two missed cleavage sites being allowed. Mass tolerances were set to 10 ppm and 0.02 Da for the precursor and fragment ions, respectively. The oxidation of methionine (+15.995 Da) was set as dynamic modification. The carbamidomethylation of cysteine (+57.021 Da) and the TMT label on lysines and the N-terminus (229.1629) were set as static modifications. False-discovery rates (FDRs) for peptide spectral matches (PSMs) were calculated and filtered using the Percolator node in Proteome Discoverer that was run with the following settings: Maximum Delta Cn 0.05, a strict target FDR of 0.01, a relaxed target FDR of 0.05 and validation based on q-value. Protein identifications were accepted when the protein FDR was below 1% and when present in at least two out of three replicate injections with at least two peptides. In addition, the final protein list was manually curated by removing uncharacterized and duplicated proteins.

### 4.7. Bioinformatic Analyses

For bioinformatic analyses, proteins with log_2_ fold change values (log2FC) ≥ 0.6 and ≤−0.6 were considered as differentially expressed (DE). The clustered heatmap of the proteins that were differentially expressed in at least one out of three treatments was generated by using CIMminer free tool (http://discover.nci.nih.gov/cimminer/ accessed on 15 March 2021) with unsupervised clustering on both axes and the following parameters: average linkage, Euclidean distance and equal width binning. Molecular function enrichment analysis was performed by using the ClueGO plug-in of Cytoscape 3.6.0 to generate a functionally grouped GO/pathway term network of enriched molecular function categories for the identified proteins based on kappa statistics [44].

## 5. Conclusions

The extensive use of synthetic fungicides over many years caused the selection of fungicide-resistant strains with serious concerns regarding environmental and food safety. The development of biological fungicides as valid non-chemical-based alternatives to synthetic agents requires a thorough understanding of the underlying mechanisms of pathogen response to treatments. 

This study represents the first attempt to compare, by means of a Tandem Mass Tag (TMT)-based high-resolution LC-MS/MS approach, the differential response to biological and synthetic antifungal agents. *P*. *digitatum*, one of the most common causes of citrus fruits postharvest spoilage, was selected as a model system for our study. The availability of genome sequence information on *P. digitatum* further corroborated the use of MS-based proteomics in the fields of fungal biology as a powerful tool for protein characterization and quantification. 

We mapped an orchestrated series of molecular events that are triggered by two toxins isolated from natural sources compared to the response elicited by the chemical fungicide thiabendazole (TBZ). Despite similarities in *P. digitatum* proteome modulation by the three anti-fungal agents, for several up- and down-regulated proteins, a different regulation was observed, suggesting the occurrence of peculiar responses to treatments. 

This study provides the first proteomic characterization of these changes and expands the existing knowledge of antifungal mechanisms of natural agents compared to chemical fungicides, paving the way for the development of new control strategies.

## Figures and Tables

**Table 1:** Proteins differentially regulated identified by high-resolution nanoLC-MS/MS in *P. digitatum* following treatments with the toxins α-sarcin and beetin 27 (BE27) and the fungicide thiabendazole (TBZ). CTR, Untreated samples. A total of 49 out of 1709 proteins were found to be differentially expressed (0.6 ≥ FC ≥ 1.5) in treated samples with respect to untreated samples.

Classification	AC	Description	α-Sarcin vs. CTR	BE27 vs. CTR	TBZ vs. CTR	Cov	#Pep	#PSM
Cell wall morphogenesis	K9GB36	GPI-anchored membrane protein	2.06	2.22	1.22	21	4	10
	K9FX15	Hydrophobin	1.99	2.73	1.76	69	5	66
	K9GEV9	C-4 methyl sterol oxidase Erg25, putative	0.71	0.64	0.82	11	3	5
	K9FUC4	Extracellular serine-threonine rich protein	1.55	2.21	1.67	7	4	10
CWDEs	K9GKS7	Pectinesterase	1.72	1.81	2.17	15	5	13
	K4MNE9	Pectate lyase	1.67	2.4	1.59	42	8	45
	K9FNZ8	Endo-polygalacturonase	1.57	1.17	0.88	19	6	21
	K9GF95	Beta-1,6-glucanase Neg1, putative	1.55	1.57	1.24	13	6	25
	K9FUA5	Arabinan endo-1,5-alpha-L-arabinosidase	1.28	1.46	1.69	6	2	3
	K4MQ21	Putative pectin lyase 2	1.06	1.16	1.68	20	4	11
	K9GKW3	Glucanase	0.76	0.62	1.11	4	2	3
Metabolic processes	K9FUV7	4-amino-5-hydroxymethyl-2-methylpyrimidine phosphate synthase	1.76	2	1.46	60	18	241
	K9G6C0	Alpha-acetolactate decarboxylase	1.7	1.22	1.64	49	12	63
	K9FLM4	Pyruvate decarboxylase	1.62	1.61	1.6	69	30	699
	K9GAM1	Terpene cyclase	1.6	1.11	1.02	41	12	60
	K9G422	Fe-containing alcohol dehydrogenase, putative	1.47	2.12	1.66	17	8	52
	K9FGJ2	3-hydroxy-3-methylglutaryl coenzyme A synthase	1.33	1.16	1.63	28	12	62
	K9GLK6	GNAT family acetyltransferase, putative	1.33	1.6	1.58	25	4	18
	K9GD10	Glutaminase GtaA	1.3	1.54	1.68	12	5	9
	K9F8A6	Pyridoxal kinase	1.26	1.53	1.05	9	2	4
	K9GVY3	Thiamine thiazole synthase	1.07	1.06	1.88	27	9	35
	K9FRK3	1-aminocyclopropane-1-carboxylate deaminase, putative	0.92	1.9	1.41	18	5	9
	K9G2P2	Aldose 1-epimerase, putative	0.66	0.72	0.82	6	3	3
	K9FYG6	Zinc-binding alcohol dehydrogenase, putative	0.63	0.65	0.59	37	10	48
	K9FWC7	Glutamate decarboxylase	0.59	0.54	0.64	52	25	125
	K9H784	Alcohol dehydrogenase 1	0.53	0.51	0.72	50	15	124
	K9FJ71	Mannitol-1-phosphate 5-dehydrogenase	0.49	0.53	1	25	8	12
Other	K9FX00	DUF5353 domain-containing protein	2.79	2.06	1.3	10	2	3
	K9GTK7	Ubiquitin-protein ligase Ufd4, putative	2.32	2.39	0.97	2	3	5
	K9G185	U3 small nucleolar RNA-associated protein 10	1.63	1.35	1.12	1	2	3
	K9G4M0	Extracellular protein, putative	1.53	2.21	1.71	6	2	3
	K9GB66	FAD-binding PCMH-type domain-containing protein	1.33	1.66	1.74	41	17	115
	K9GEC9	AMP-binding enzyme, putative	0.86	1	1.99	4	2	3
	K9F863	Purple acid phosphatase	0.57	0.56	0.95	16	5	10
	K9FDZ3	C2H2-type domain-containing protein	1.53	1.21	1.11	11	5	9
Protease	K9GB19	Trypsin-like serine protease, putative	1.59	2.05	1.98	27	6	73
	K9FI66	Tripeptidyl-peptidase sed2	1.3	1.64	1.47	29	10	37
	K9GFK6	Protease S8 tripeptidyl peptidase I, putative	0.66	0.67	1.44	3	3	4
Oxidative stress response	K9GPG9	Glyoxalase family protein	1.6	1.92	1.25	6	3	5
	K9G667	Glutaredoxin-like protein	0.55	0.45	0.88	28	4	11
	K9G5U5	Catalase	1.16	1.23	1.6	27	15	35
RNA binding	K9FAA5	Nuclear mRNA splicing factor-associated protein, putative	0.89	1.7	1.03	3	2	3
	K9GW78	Signal recognition particle 19 kDa protein, putative	0.67	0.65	0.85	7	2	4
	K9FUM5	DEAH-box RNA helicase (Dhr1), putative	0.65	0.62	0.89	2	3	4
Stress response and transporters	K9FZM3	Photolyase/cryptochrome alpha/beta domain-containing protein	1.27	1.3	2.13	3	2	3
	K9FGI7	Antifungal protein Afp	1.06	0.6	1.18	23	3	9
	K9F4E2	ZIP Zinc transporter, putative	0.7	0.63	0.69	7	3	5
	K9H0P1	MFS monosaccharide transporter, putative	0.66	0.64	0.71	5	3	10
	K9FBR5	SurE domain-containing protein	0.62	0.74	1.12	26	6	20

## Data Availability

Data are available upon request; please contact the contributing authors.

## Supplementary Materials

Supplementary materials can be found at https://www.mdpi.com/article/10.3390/ijms23020680/s1.

## Abbreviations

BE27	Beetin 27
CWDEs	Cell Wall-Degrading Enzymes
MS	Mass Spectrometry
RIPs	Ribosome-Inactivating Proteins
SRL	Sarcin Ricin Loop
TBZ	Thiabendazole
TMT	Tandem Mass Tag
