# Supplementary material for: Deciphering Molecular Determinants Underlying Penicillium digitatum’s Response to Biological and Chemical Antifungal Agents by Tandem Mass Tag (TMT)-Based High-Resolution LC-MS/MS
Source: Int J Mol Sci. 2022 Jan 8;23(2):680. doi: 10.3390/ijms23020680 (PMC8775614; doi:10.3390/ijms23020680)

Supplementary Material

Deciphering molecular determinants underlying *Penicillium digitatum* response to biological and chemical antifungal agents by Tandem Mass Tag (TMT)-based high-resolution LC-MS/MS

Lucía Citores[1‡], Mariangela Valletta[2‡], Vikram Pratap Singh[2], Paolo Vincenzo Pedone[2], Rosario Iglesias[1], José M. Ferreras[1], Angela Chambery[§2*], Rosita Russo[§2*]

[1]Department of Department of Biochemistry and Molecular Biology and Physiology, Faculty of Sciences, University of Valladolid, E-47011 Valladolid, Spain; lucia.citores@uva.es (L.C.); riglesia@bio.uva.es (R.I.); josemiguel.ferreras@uva.es (J.M.F.)
[2]Department of Environmental, Biological and Pharmaceutical Sciences and Technologies, University of Campania Luigi Vanvitelli, Caserta, Italy; mariangela.valletta@unicampania.it (M.V.); vikrampratap.singh@unicampania.it (V.P.S.); paolovincenzo.pedone@unicampania.it (P.V.P.); angela.chambery@unicampania.it (A.C.); rosita.russo@unicampania.it (R.R.)

[*]Correspondence: angela.chambery@unicampania.it; Tel.: +39 (0)823 274583; rosita.russo@unicampania.it; Tel.: +39 (0)823 274535;

[‡] These authors share equal first authorship
[§] These authors share equal senior authorship

Figure S1. Bar charts reporting the counts of peptide groups abundances versus CV% values for α-sarcin-, BE27- and TBZ -treated and untreated (CTR) *P. digitatum* samples.

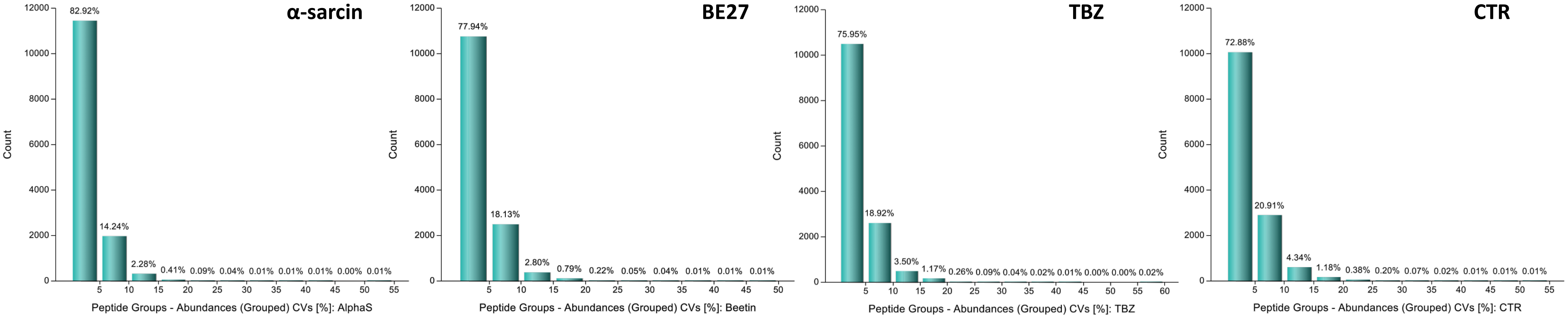

Figure S2. Volcano plots obtained from TMT-based quantitative proteomics analysis showing −Log10 p-values versus Log2 fold-change values of TBZ, BE27 and α-sarcin treatments in *P. digitatum* with respect to control (CTR). Plots are referred to unfiltered datasets. Identified proteins with no changes in their regulation levels are reported in grey. Up- and down-regulated proteins (fold change ≥ 1.5) are indicated in red and green, respectively.

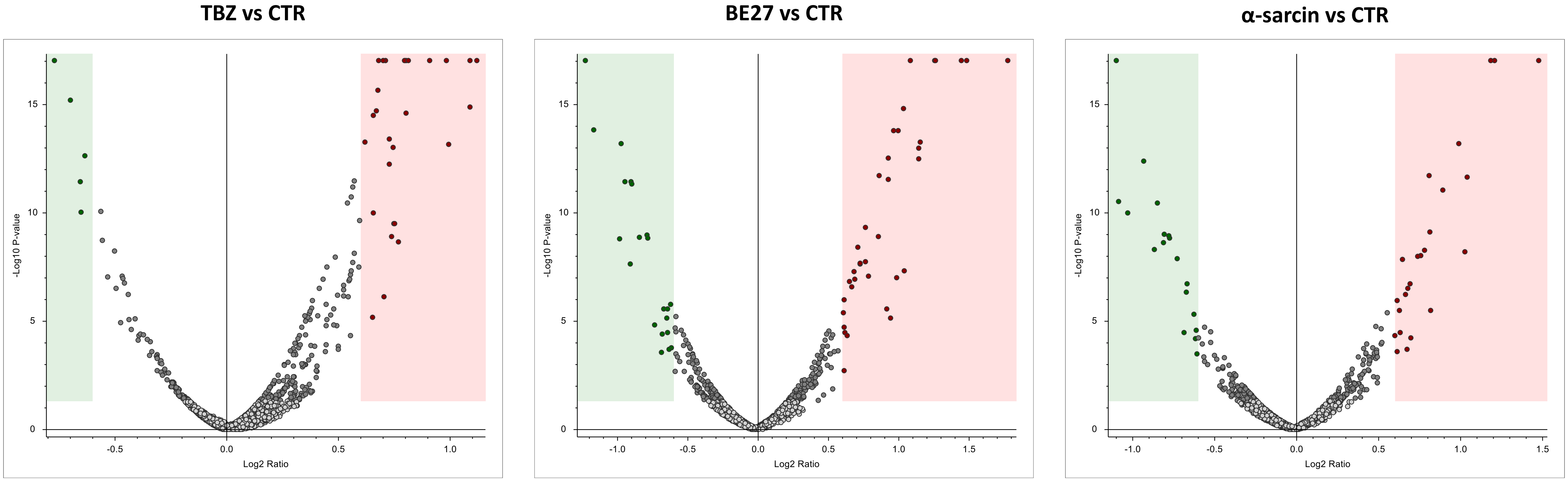

Supplement: Supplementary file 1 [file ijms-23-00680-s001.zip › Supplementary Material Figures S1 and S2.pdf]
